# Quantifying epileptic networks: every data point brings us a step closer to an optimized surgery

**DOI:** 10.1093/braincomms/fcae349

**Published:** 2024-10-04

**Authors:** John Thomas, Kassem Jaber, Birgit Frauscher

**Affiliations:** Department of Biomedical Engineering, Duke Pratt School of Engineering, Durham, NC, USA; Department of Biomedical Engineering, Duke Pratt School of Engineering, Durham, NC, USA; Department of Biomedical Engineering, Duke Pratt School of Engineering, Durham, NC, USA; Department of Neurology, Duke University Medical Center, Durham, NC, USA

## Abstract

This scientific commentary refers to ‘The sixth sense: how much does interictal intracranial EEG add to determining the focality of epileptic networks?’, by Gallagher *et al*. (https://doi.org/10.1093/braincomms/fcae320).

**This scientific commentary refers to ‘The sixth sense: how much does interictal intracranial EEG add to determining the focality of epileptic networks?’, by Gallagher *et al*. (** https://doi.org/10.1093/braincomms/fcae320 **).**

Surgery using invasive electroencephalography (iEEG) offers an effective treatment for people with drug-resistant focal epilepsy. IEEG is used to measure the epileptic network and to provide a precise location in the brain to intervene, thereby reducing functional deficits. Despite significant technological advancements, the success rate of epilepsy surgery remains stagnant at 60%. Therefore, developing personalized models is crucial to accurately identify patients who will benefit from iEEG and subsequently from an iEEG-informed surgery. This approach will also be beneficial in patient cases with a low likelihood of achieving seizure freedom, allowing for the earlier initiation of alternative treatment options such as neuromodulation.

Recently, in *Brain Communications*, Gallagher and colleagues have introduced a simple, easy-to-implement approach that integrates the spatial distribution of iEEG electrodes and abnormality. This method helps to determine whether the epileptic network is focal or widespread, thereby aiding in the decision to proceed with intervention following an iEEG monitoring.^1^ The study was performed on a large cohort of 101 patients from a single centre, of which 65 had focal epilepsy. The classification paradigm was designed to differentiate between focal and non-focal cases and between surgically treated and device-treated patients. However, the number of device-treated cases was relatively small, with 22 patients, which may result in a lack of statistical power.

The study evaluates the 5-SENSE score^2^ and two computational measures, namely, the ‘implant distance’ and the ‘abnormality distance’. The 5-SENSE score assesses the focality of epilepsy using non-invasive features and guides decisions on whether to proceed with an iEEG. Conversely, the implant distance and the abnormality distance are post-implantation metrics that evaluate the focality of the epileptic network with respect to the spatial placement of iEEG electrodes. The higher the distance value, the more widespread the epileptic focus.

Each of the 14 features investigated (the 5-SENSE score, implant distance, abnormality in six spectral bands and six connectivity measures) demonstrated varying degrees of separation between focal and non-focal epilepsy patients. The 5-SENSE score achieved the highest effect size, followed by abnormalities in the gamma band power. These results align with the following findings:

- The 5-SENSE score can assist clinicians in decision-making by identifying patients where iEEG is unlikely to measure a focal generator.^2^
- Abnormal gamma activity, particularly those preceding spikes, can serve as a biomarker to guide epilepsy surgery.^3,4^
- Incorporating the spatial distribution of iEEG electrodes with abnormal gamma activity can help determine whether the implantation of iEEG is adequate to identify the seizure focus.^5^

The major contribution of the manuscript is that the new features offered complementary information to the 5-SENSE score. By combining the three features, namely, the 5-SENSE score, implant distance and abnormality distance into a model, the authors achieved a statistically superior area under the receiver operating characteristic curve (AUC = 0.79) than the 5-SENSE score. This model was also able to distinguish between seizure-free and non–seizure-free post-operative outcomes, with an AUC of 0.70. Another important aspect is that all three measures generalized well to subdural and stereo-EEG electrodes. This is interesting given that the 5-SENSE score was originally developed and validated for stereo-EEG. These findings have significant clinical implications. This model can estimate the focality of the epileptic network, helping to identify candidates who may benefit from surgical interventions following an iEEG implantation. Conversely, it can also identify patients with distributed or multifocal epileptic networks, who are less likely to achieve a seizure-free outcome, thereby providing early recommendations against surgical interventions.

The study raises four potential challenges (Fig. 1). First, iEEG has limited brain coverage, potentially leading to misidentification of the ‘true’ epileptic focus. It is crucial to assess the adequacy of the implantation before using the data for feature analysis or making informed decisions about whether to proceed with a surgical intervention. In the current study, we are unable to evaluate if mis-sampling may have influenced the results. Second, four distinct categories of patients emerge from the study (Fig. 2). In Groups 1 and 4, where the 5-SENSE score aligns with the proposed model, the model serves to corroborate the results. In Group 2, the 5-SENSE predicted focality, but the model suggested otherwise, indicating that surgery might lead to poor outcomes. This discrepancy could be explained by the possibility of mis-sampling in iEEG implantation. Therefore, an evaluation of the implantation scheme is required to assess the model’s performance. Group 3 presents a controversy: if 5-SENSE fails to identify focality, should we proceed with an iEEG implantation? The answer from this study and the 5-SENSE validation study is yes and will depend on the individual situation [specificity in the validation cohort was 76% (95% CI, 67.5–84.0%)] and the goal of implantation.^2^ The 5-SENSE score was designed to assess if an iEEG will be beneficial and not to predict surgical outcomes. A *post hoc* evaluation of these four groups in relation to surgery outcomes would have been beneficial, particularly for Groups 2 and 3, where discrepancies were observed between the model and the 5-SENSE score. This evaluation could have revealed unique phenotypes of patients, allowing for more personalized treatment approaches.

**Figure 1:**
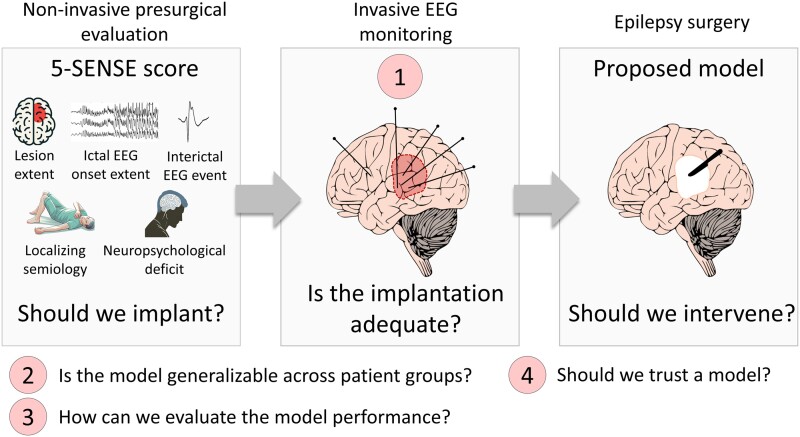
**Potential challenges in the study by Gallagher *et al*.** ^1^ The 5-SENSE score predicts whether the iEEG will reveal a focal seizure-onset zone. This decision is based on a comprehensive Phase 1 evaluation, which includes findings from seizure semiology, scalp video EEG, structural magnetic resonance imaging, and neuropsychological testing. The proposed model predicts whether we should intervene, given the iEEG implantation results. The four potential challenges are numbered from 1 to 4. This image was created with https://freesvg.org/vector-drawing-of-a-human-brain-with-cerebellum.

**Figure 2:**
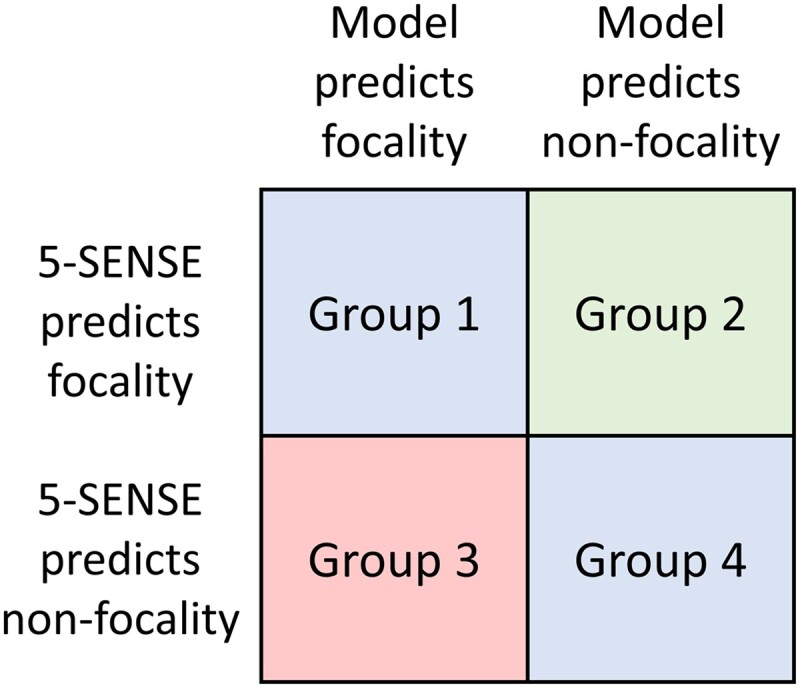
**The four patient groups based on agreement between the 5-SENSE score and the proposed model in the study by Gallagher *et al*.** ^1^

Third, there is a more general issue: which metric should we use to evaluate predictive models for epilepsy surgery? The AUC does not provide information about the spatial distribution of model errors. It treats all misclassifications equally, but the costs and benefits of different types of errors can vary in real-world scenarios. For example, in the literature, biomarkers or models might be biased towards patients with focal cortical dysplasia or hippocampal sclerosis, as these groups have a higher proportion of seizure-free outcomes. Furthermore, AUC represents a trade-off between sensitivity and specificity. High sensitivity is desirable for screening, whereas, for surgical decisions, high specificity is crucial, adhering to the principle of ‘primum non nocere’.

Finally, scores and models can only guide clinicians. In specific scenarios, even non-focal epilepsy (defined as lobar, multi-lobar, multifocal, or missed seizure-onset zone in the 5-SENSE study^2^) can be treated surgically, such as in the case of temporal plus epilepsy, and patients may still achieve seizure freedom with multi-lobar resections. The final treatment decision always rests with the clinician. Given the limited number of epilepsy surgery cases at a single centre, learning from past cases of many centres and formulating an optimized treatment plan might be a helpful tool for the future.^6^

Within the context of this study, we have several recommendations. First, we suggest assessing the quality of iEEG implantation before analysing iEEG features or models for epilepsy surgery. Second, we encourage performing a *post hoc* analysis to evaluate whether the feature or model may be biased towards a particular subgroup of patients. Next, while reporting performance measures, including multiple metrics such as AUC, the area under the precision–recall curve, sensitivity, specificity and the F1 score may be beneficial. This comprehensive reporting will facilitate comparisons with both future and existing studies. Finally, we should conduct prospective and multicentric clinical trials to evaluate promising models and scores for optimizing epilepsy surgery.

## Limitations

Despite its promising findings, this study has a few limitations. As a single-centre retrospective study, the generalizability of these results to other centres remains uncertain. The rationale for iEEG implementation and the associated terminology can vary across countries and levels of expertise.^7^ Consequently, the criteria used for implantation may differ from centre to centre, which could necessitate redefinitions of both implant distance and abnormality distance. Therefore, validating the proposed measures and model across data from multiple centres and implantation schemes is necessary.

The authors utilized a simple measure of deviation from normative spectral and connectivity measures. Comparing these findings with existing literature on iEEG biomarkers such as spikes, spike-gamma/ripple, spike propagation and even the aperiodic component of the EEG spectrum^3,4,8-11^ could potentially strengthen the results. This approach could provide a quantifiable and/or visual definition of what the abnormality distance specifically captures. Additionally, the use of awake iEEG increases the likelihood of artefacts, particularly in the high-frequency band and in superficial contacts compared to sleep iEEG, which may further make the interpretation of results challenging.

## Conclusion

Gallagher *et al.*^1^’s study represents a step forward in developing quantitative tools to guide epilepsy surgery. By demonstrating that spatial measures of interictal iEEG abnormalities can guide decisions on whether to proceed with surgical intervention, this study offers a framework for more objective, data-driven surgical decision-making. Future studies should aim to validate the proposed model across multiple centres and larger patient cohorts. This will be crucial for fine-tuning the model to enhance its generalizability and maximize its clinical utility. In addition, incorporating other quantitative biomarkers could further improve the accuracy of focality predictions. Ultimately, adopting quantitative models in epilepsy surgery could enhance outcomes by equipping clinicians with the tools needed for more informed and personalized treatment decisions.

## Data Availability

No new data were generated or analysed in support of this research.
